# Predictors of performance on the Reading the Mind in the Eyes Test

**DOI:** 10.1371/journal.pone.0235529

**Published:** 2020-07-23

**Authors:** Clare M. Eddy, Peter C. Hansen

**Affiliations:** 1 National Centre for Mental Health, BSMHFT, Birmingham, United Kingdom; 2 Institute of Clinical Sciences, College of Medical and Dental Sciences, University of Birmingham, Birmingham, United Kingdom; 3 Centre for Human Brain Health and School of Psychology, College of Life and Environmental Sciences, University of Birmingham, Birmingham, United Kingdom; Univdersity Hospital of TübingenUniversitatsklinikum Tubingen, GERMANY

## Abstract

We explored factors associated with performance on the Reading the Mind in the Eyes Test (RMET). 180 undergraduate students completed the human RMET requiring forced-choice mental state judgment; a control human Age Eyes Test (AET) requiring age judgment; a Cat Eyes Test (CET) requiring mental state judgment; and measures of executive function, empathy and psychopathology. Versions of the CET and AET were created that matched the RMET for difficulty (accuracy 71%). RMET and CET performance were strongly correlated after accounting for AET performance. Working memory, schizotypal personality and empathy predicted RMET accuracy but not CET scores. Liking dogs predicted higher accuracy on all eyes tasks, whereas liking cats predicted greater mentalizing but reduced emotional expression. Importantly, we replicated our core findings relating to accuracy and correlations between the CET and RMET in a second sample of 228 students. In conclusion, people can apply similar skills when interpreting cat and human expressions. As RMET and CET performance were found to be differentially affected by executive function and psychopathology, the use of social cognitive measures featuring non-human animals may be of particular use in future clinical research.

## Introduction

The Reading the Mind in the Eyes Test (RMET) [1] assesses the ability to recognise complex mental states as expressed by human eyes. Participants pick one of four options (e.g. puzzled, nervous, insisting or contemplative) which they think best describes what the person in each photograph is thinking or feeling. Correct answers are based on majority responses from a number of expert judges [1] from a healthy population. Many previous studies have explored the influence of neurological and psychiatric disorders on performance. For example, patients with autism [1, 2], Parkinson’s disease [3], Huntington’s disease [4, 5], Tourette syndrome [6] and schizophrenia [7] have been shown to offer fewer correct (conventional) responses when compared to a healthy control group.

The RMET could evoke higher cognitive reasoning about mental states (theory of mind), recognition of visual cues to emotion, and/or empathy. In addition to clinical symptoms, general cognitive or perceptual skills may influence performance. Previous fMRI studies have revealed activation in cortical regions and underlying structures such as superior temporal sulcus, inferior frontal gyrus, medial prefrontal cortex, hippocampus and cerebellum during the RMET [8–11]. It is thought that RMET judgments reflect a fast, automatic process [1], and response consensus implies that the RMET measures a common human ability or collection of skills. Given that the processes involved in the eyes task are still poorly understood, the current study aimed to explore factors related to recognition of mental states from eyes, using the RMET and two other tasks: the same behavioural task with different stimuli, and the same stimuli requiring an alternative behavioural response.

The Cat Eyes Test (CET) was created as a comparison measure for the current study, and required participants to select mental states to match pairs of cat eyes. Cats were selected due to frequency of exposure to humans, and because there were many images freely available online for developing this task. Cat eyes could be perceived to depict complex mental states given that the form of the human face is similar in many ways to other mammals [12]. It could be suggested that the CET will invoke anthropomorphism: a cognitive bias whereby people spontaneously ascribe human characteristics to a non-human agent [13]. However, while most previous studies have investigated spontaneous attribution of emotions to pets [14], the current study required participants to make a forced choice about the appropriate mental state, rather than assessing spontaneous mental state attribution. If healthy participants reach a common interpretation of each cat’s mental state (as for the human RMET) this may imply cues to real emotion within the images that could approximate human expressions, or that the skills involved in mental state attribution during the RMET are not specific to human stimuli.

An advantage of developing the CET relates to previous studies in autistic spectrum disorder (ASD). Hypoactivation of the fusiform gyrus is seen in ASD in response to human faces but not animal faces [15], and while typically developing children spend more time looking at human eyes than the eyes of animals including cats, children with ASD spend more time looking at animal eyes [16]. Therefore a new mental state recognition measure involving animal facial features rather than human features could offer further insight into the skills of those with ASD.

The second task was the age eyes task (AET), which requires judgments about the physical state of the human RMET stimuli. The AET is of a similar difficulty to the original RMET but elicits less limbic activity than the RMET in healthy participants [17], perhaps drawing upon executive function and autobiographical memory rather than emotional processing. The current study included two measures of executive function, predicting that executive functions would be more closely related to performance on the AET than the RMET or CET.

Previous studies have linked RMET performance to the Empathy Quotient (EQ) [18], the Interpersonal Reactivity Index (IRI) [19, 20] which explores self-reported perspective taking, and the Toronto Alexithymia Scale (TAS-20) [21, 22], which measures reflection on and communication about one’s own emotions. Lyvers, Kohlsdorf, Edwards & Thorberg [23] found that high alexithymia in students predicted low empathy and poor RMET performance. Difficulties in interpreting one’s own emotions could therefore impair recognition of emotions in others. One study in undergraduates [24] found that low EQ scores were associated with high alexithymia and low RMET accuracy. Demers and Koven [25] report that in healthy adults RMET scores are positively correlated with emotional empathy, and negatively correlated with alexithymia. We included the EQ, IRI and TAS-20 in the current study, hypothesising that lower accuracy on the RMET and CET would correlate with lower IRI scores and higher TAS scores.

Participants completed three other scales to explore eyes task performance in relation to clinical symptoms. The first was the Schizotypal Personality Scale (SPQ) [26], as Irani et al. [27] found that high levels of schizotypal personality traits (e.g. social anxiety, constricted affect) were linked to poorer RMET performance. The Obsessive-Compulsive Inventory (OCI-R) [28] was included because of there being few previous studies into the relationship between these symptoms and social cognition, despite sub-threshold obsessive and compulsive traits being common within healthy populations [29]. Finally, we included the revised Social Anhedonia Scale (rSAS) [30], as social anhedonia (reduced pleasure from social interactions) can be linked to both autism and alexithymia [31]. We expected high scores on these clinical scales would be related to lower accuracy on the RMET and CET.

In summary, we explored attribution of mental states on the human RMET as compared to a comparison task using cat eyes, and a matched control task involving age judgment of RMET stimuli. We selected cat stimuli as this is a mammal that is familiar to humans and we wanted to use non-human stimuli given that evidence from previous studies suggests this could be a useful comparison to tasks involving human stimuli, perhaps especially in clinical groups [15, 16]. To offer insight into factors influencing performance on the three eyes tasks, we included measures of empathy, executive functions and specific clinical symptoms. We also included a pet questionnaire to offer further insight into responses on the CET, as exposure to animals or pets may be linked to anthropomorphising and in turn emotion attribution on tasks involving animal stimuli [14]. In addition, we conducted psychometric analysis on the CET and AET, aiming to determine whether it was possible to use these measures as control tasks for the RMET, matched for accuracy.

## Materials and methods

### Participants

This study was approved by the University of Birmingham Research Ethics Committee and all participants gave written informed consent. Participants were 180 undergraduate Psychology students (details after exclusions below) without existing psychiatric/neurological diagnoses or cat phobia. We recruited as many volunteers as possible, who received course credit for participation.

### Procedure

Basic instructions were given for each task before completion by the participant, in the order: Digit Ordering Test-Adapted (DOT-A), Trail Making Test (TMT), pet questionnaire, IRI, TAS-20, SPQ, OCI-R, EQ, rSAS. Participants then completed the three computerised eyes tasks (two runs of each), presented using Presentation (Neurobehavioral Systems Inc.) software. The order of administration of these three tasks was counterbalanced across participants and stimuli within each were in randomised order.

### Tasks

#### RMET

**Fig 1:**
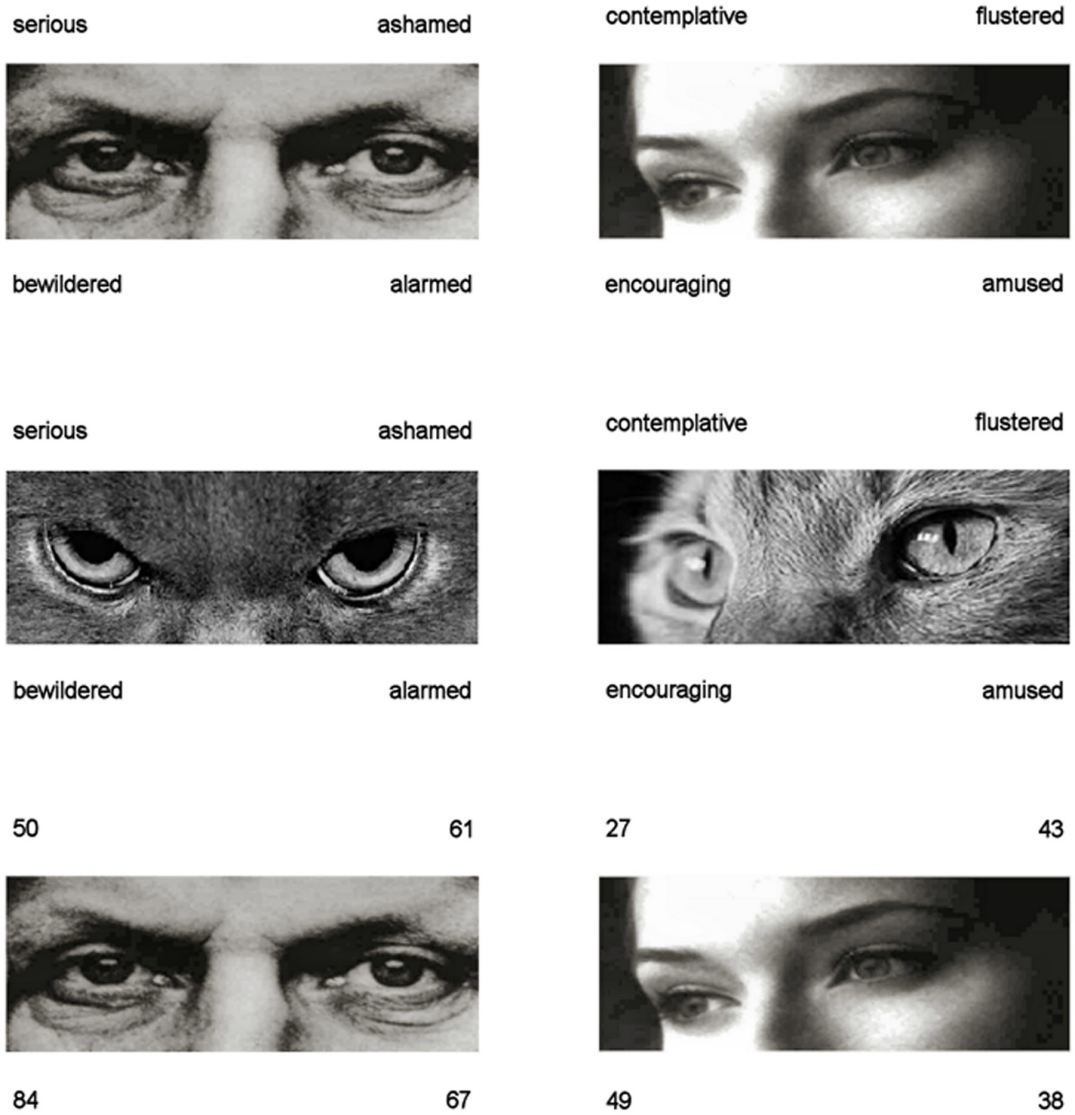
Example stimuli from all three eyes tasks (Reading the Mind in the Eyes Test; Cat Eyes Test; Age Eyes Test). Correct answers for trials shown are ‘serious’ and ‘contemplative’. RMET stimuli freely available online (https://www.autismresearchcentre.com/arc_tests) and cat images within public domain/under CC license 2019 (https://www.maxpixel.net/Eyes-Animal-Pet-Blue-Eye-Cats-Eyes-View-Cat-1285634; https://www.flickr.com/photos/felinest/4394881615).

The RMET contains 36 test trials plus one practice item (available from https://www.autismresearchcentre.com/arc_tests). Stimuli are photographs of human eyes, surrounded by four mental state options (Fig 1). Instructions (1) require the participant to consider the options (a glossary is available) and select the option they think best matches what the person in the photograph is thinking or feeling. There is no time limit. Evidence of task validity comes from the ability of this task to differentiate between individuals with ASD and typically developing individuals (e.g. 1). The RMET has reasonably good test-retest reliability [24].

The RMET commenced with onscreen instructions to view the stimulus and pick ‘the word that best describes what the person in the image is thinking or feeling’. Images were approximately 28cm x 9cm high (24" monitor; resolution 1024 x 768), with response options in Arial 22 point (approximately 1cm high) outside the corners of the image, mapped to the numeric keypad [1, 3, 7, 9]. The first trial was initiated via pressing the spacebar. There was no time limit, and a response initiated the next trial.

#### CET

The CET was developed by one experimenter (CME) selecting online images (freely available for reuse) to match the original set of RMET expressions/answers, taking into account visual similarity (e.g. gaze direction) where possible. The testing procedure was equivalent to the human RMET i.e. participants were asked to select the word they think best matched what the cat in the image was thinking or feeling (see Fig 1).

#### AET

The AET (Fig 1) used the same stimuli as the original RMET, and was devised previously [17]. Instructions and administration of the AET were equivalent to the other eyes tasks, but asked participants to pick the number that best matched the age of the eyes.

#### Pet questionnaire

The pet questionnaire asked if respondents had a ‘pet now’ or a ‘pet previously’ (Y/N). Participants were also asked to rate ‘liking cats’ and ‘liking dogs’ on a 7-point Likert scale from -3 (I hate) to +3 (I love).

#### DOT-A

Participants heard strings of mixed up digits (e.g. 4-8-1-3) read out by the experimenter (a pair of strings individually presented for each length of 3 to 8 digits). After each they were required to speak the digits aloud in ascending order. Testing ended when 2 strings of the same length were answered incorrectly, with half a point deducted from the maximum working memory span for one string of a pair answered correctly [32], possible range 2.5–8 digits.

#### TMT

The baseline condition required participants to draw lines accurately connecting a series of numbered circles (1–25) as quickly as possible, keeping the pen on the page. The test condition contained numbers (1–13) and letters (A-L) and participants had to swap between categories i.e. join 1-A, A-2, 2-B etc. The time difference to complete conditions (test–baseline) was used as an index of interference when attention shifting.

#### IRI

The IRI [19, 20] contains 4 subscales each with 7 items (scored from 1–5; total score range 28–140; subscales 7–28). Perspective taking (PT) assesses the tendency to adopt other people’s points of view, and empathic concern (EC) addresses feelings of warmth and consideration towards others. High scores for personal distress (PD) indicate greater negative emotion when around other people in distress and the fantasy subscale measures the propensity to imagine and relate to characters in books and films.

#### TAS-20

This alexithymia scale (possible range 20–100) demonstrates good reliability and validity [21, 22]. There are three subscales: difficulty identifying feelings (DIF e.g. “I have feelings that I can’t quite identify”); difficulty describing feelings (DDF e.g. “It is difficult for me to find the right word for my feelings”) and externally oriented thinking (EOT e.g. “I prefer to just let things happen rather than to understand why they turned out that way”). The cut-off for non-alexithymia is below 51 and for probable alexithymia it is 61 or above.

#### SPQ

The 74 SPQ items are grouped into nine subscales (each item scores 0/1): constricted affect, no close friends (NCF), excessive social anxiety (ESA), unusual perceptual experiences (UPE), odd speech, odd beliefs or magical thinking, suspiciousness (SUS), ideas of reference, and odd/eccentric behaviours (OEB). Previous studies report good internal consistency, test-retest reliability and validity [26]. Three major factors have also been identified [27]: cognitive perceptual (IOR, OBMT, UPE, SUS), social interpersonal (ESA, NCF, CA) and disorganization (OEB, OS).

#### OCI-R

This scale [29] contains 18 items such as “I check things more often than necessary” and “I find it difficult to control my own thoughts”; responded to on a 5-point Likert scale (0–4) from ‘not at all’ to ‘extremely’. Scores can range from 0 to 72, and the authors recommend a cut-off of 21 to indicate likely OCD.

#### EQ

The EQ [18] contains 40 empathy questions and 20 fillers. Responses are scored 0–2, resulting in a possible score of 0–80. EQ scores can be inversely correlated with ASD [18].

#### RSAS

The revised rSAS [30] contains 40 items and assesses social withdrawal and lack of pleasure from social relationships e.g. “A car ride is much more enjoyable if someone is with me”; “Having close friends is not as important as some people say”. Suggested cut-off score is 16 for females and 20 for males (higher scores indicate greater social anhedonia).

### Statistical processing

Two participants were excluded (accuracy below chance n = 1; fast RT/low accuracy n = 1) and a full data set on the eyes tasks was not available for a further two participants due to technical problems. A further four participants had incomplete data on one or two of the behavioural scales but were included after imputation of missing values based on group mean [33]. Therefore data from 176 participants was used for analysis (16 males and 160 females, mean age 19.65 years (SD = 1.29; range = 18.23–32.82). Individual outliers per task were removed (1.3% of the data) based on a reaction time (RT) ≤200msec, or >3 times SD + mean RT.

First we explored response consensus (i.e. accuracy) and psychometric properties, followed by partial correlations between eyes tasks. We then ran regression analyses with eyes task scores as DVs and all other measures as IVs followed by post-hoc analysis on any identified relationships.

## Results

### Eyes task accuracy (consensus)

Responses to each eyes task are shown in Table 1. We used the majority response across the whole sample as a correct response for the CET, and also the AET, and the correct answers provided by Baron-Cohen et al. [1] for the RMET. In order to compare the three tasks when exploring factors that influenced eyes task performance, we first needed to match for difficulty. We therefore selected subsets of CET and AET stimuli so that none of the three eyes tasks significantly differed in terms of accuracy. This resulted in a subset of 18 trials for the CET, and 16 trials for the AET. Overall accuracy was ~71% for each eyes task: 70.77% (SE = 0.69%) for the RMET, 70.57% (SE = 0.81%) for the CET, and 70.51% (SE = 0.72%) for the AET. A logistic regression mixed effects model (DV: individual trial accuracy correct/incorrect; fixed factors: gender, run and condition; random effect: Participant ID) was used to make inferences about the wider population beyond the sample. This showed no significant effect of gender, run or task, but there was a significant interaction between run and condition (χ^2^(2) = 7.05, p = .03). Accuracy was greater for the age task on the first run, but this effect was not seen for the RMET or CET. Post hoc comparisons with Tukey correction confirmed there were no significant differences between RMET versus AET (z = -0.358, p = .932; 95% CI); RMET versus CET (z = 0.300, p = .952; 95% CI); or AET versus CET (z = -.061, p = .998; 95% CI). For some individual trials, greater accuracy was reached for the CET than the RMET (Table 1; S1 Fig).

**Table 1:** Eyes tasks consensus: Percentage of participants selecting each response option per item.

	Human Age Eyes Test (AET)				Human Eyes Test (RMET, mental state)				Cat Eyes Test (CET, mental state)			
Item	1	2	3	4	1	2	3	4	1	2	3	4
1	17.19	10.60	2.29	**69.91**	59.89*	25.21	6.30	8.60	14.33	20.92	26.36	38.40
2	5.73	0.00	**93.98**	0.29	11.68	84.90*	0.85	2.56	14.77	**83.24**	0.85	1.14
3	3.79	**56.27**	31.49	8.45	2.87	8.33	69.54*	19.25	5.23	8.43	**64.83**	21.51
4	41.74	31.30	21.74	5.22	2.02	82.95*	3.18	11.85	1.44	**88.22**	3.45	6.90
5	2.91	22.97	35.47	38.66	7.12	8.83	82.34*	1.71	4.00	1.14	**79.43**	15.43
6	4.91	**70.23**	1.16	23.70	5.73	60.17*	29.23	4.87	6.07	**61.27**	24.28	8.38
7	31.12	9.51	7.78	51.59	7.80	19.65	55.49*	17.05	11.30	28.41	**57.68**	2.61
8	36.47	37.61	10.26	15.67	68.41*	16.81	13.62	1.16	30.95	18.34	46.70	4.01
9	1.16	22.38	8.72	**67.73**	9.77	5.75	4.60	79.89*	4.05	9.83	31.50	54.62
10	54.57	0.86	42.00	2.57	60.63*	22.41	8.62	8.33	**58.00**	19.43	5.43	17.14
11	2.85	31.62	0.57	**64.96**	9.20	9.77	78.45*	2.59	38.33	6.34	53.03	2.31
12	2.33	35.28	44.90	17.49	12.93	4.02	78.16*	4.89	16.09	1.44	**73.85**	8.62
13	36.57	1.71	37.71	24.00	7.45	63.04*	5.44	24.07	12.89	**62.75**	13.75	10.60
14	**57.80**	26.01	4.91	11.27	16.33	16.03	4.66	62.97*	52.59	8.05	2.87	36.49
15	**88.86**	0.57	2.00	8.57	76.66*	4.90	10.66	7.78	**70.77**	7.45	13.18	8.60
16	**58.50**	24.21	14.99	2.31	7.80	67.63*	7.23	17.34	41.09	46.84	2.59	9.48
17	55.91	5.76	16.71	21.61	71.10*	13.87	9.25	5.78	33.24	28.32	27.17	11.27
18	1.15	44.70	6.88	47.28	64.08*	13.51	4.31	18.10	36.34	12.50	31.69	19.48
19	8.65	34.29	32.56	24.50	8.12	22.61	11.88	57.39*	15.16	13.12	9.91	**61.81**
20	16.00	33.43	39.14	11.43	8.57	82.00*	8.29	1.14	47.55	36.89	10.09	5.48
21	17.29	**65.99**	14.70	2.02	2.87	91.40*	4.58	1.15	5.78	46.53	21.10	26.59
22	36.23	45.80	2.61	15.36	71.55*	1.15	5.17	22.13	35.67	3.51	26.02	34.80
23	49.71	4.05	19.94	26.30	14.70	10.66	37.46*	37.18	4.87	4.30	**75.07**	15.76
24	54.15	4.87	1.43	39.54	70.72*	10.72	6.96	11.59	39.77	13.54	29.11	17.58
25	**62.00**	11.43	0.86	25.71	2.95	17.70	12.39	66.96*	26.90	7.89	17.54	47.66
26	0.28	**93.16**	5.13	1.42	10.06	5.17	68.97*	15.80	9.40	1.99	**84.05**	4.56
27	**87.71**	1.71	2.00	8.57	1.15	77.87*	11.49	9.48	4.34	**58.09**	17.34	20.23
28	33.24	52.89	3.18	10.69	65.80*	2.03	24.35	7.83	30.35	2.31	56.94	10.40
29	0.86	53.30	35.24	10.60	22.19	3.75	19.88	54.18*	15.47	4.58	**63.61**	16.33
30	0.57	6.61	37.64	55.17	2.01	92.26*	3.72	2.01	9.30	27.33	52.62	10.76
31	15.32	1.73	56.36	26.59	7.76	75.57*	4.31	12.36	10.53	52.34	3.22	33.92
32	8.31	16.91	26.65	48.14	81.53*	5.68	4.26	8.52	**80.57**	3.71	6.29	9.43
33	13.83	5.48	**57.93**	22.77	3.15	14.33	5.16	77.36*	12.25	29.63	18.80	39.32
34	2.84	5.97	**67.05**	24.15	7.20	18.16	67.44*	7.20	11.88	38.26	33.62	16.23
35	24.93	19.48	47.28	8.31	9.77	52.01*	18.68	19.54	26.50	**62.96**	6.55	3.99
36	**66.00**	23.14	9.71	1.14	3.14	2.57	90.00*	4.29	7.45	2.29	**85.10**	5.16

‘Correct’ responses for the RMET are denoted by *; **BOLD** are used in final refined subset of CET/AET.

Mean RT (seconds; collapsed across run) was 4.47s (SE = .10) for the RMET; 4.16s (SE = .08) for the CET, and 3.52s (SE = .06) for the AET. A mixed effects model (DV: RT; fixed factors: age, gender, run and task; random factor: participant ID; fixed variance weighting as a function of RT to correct for heteroscedascity) showed a significant effect of run (F(1, 24200) = 228.8, p<.0001) and task (F(2, 24200) = 260.5, p<.0001) but not for gender or age; and a significant interaction between run and task (F(2, 24200) = 39.8, p<.0001). Post hoc comparisons with Tukey correction (two tailed) showed a significant difference for AET versus RMET (t(24200) = -25.20, p<.0001, 95% CI), AET versus CET (t(24200) = -14.13, p<.0001, 95% CI), and RMET versus CET (t(24200) = 9.06, p<.0001, 95% CI). RT was longest for the RMET and shortest for the AET. Overall run 1 was slower than run 2 (t(24200) = 39.770, p<.0001, 95% CI), but the interaction was explained by this difference being most pronounced for the RMET. RT is not a standard measure for the RMET so we focus hereafter on accuracy.

### Psychometric analysis

Overall reliability using mean Cohen’s Kappa (between runs) was moderate for all eyes tasks, and slightly lower for the AET (0.540, SE = 0.014) and CET (0.532, SE = 0.016) than for the RMET (0.564, SE = 0.014). Paired t-tests indicated that RMET was significantly different to CET (t(175) = 2.35, p = .02) but the CET and AET were not (t(175) = 0.49, p = .62) and AET and RMET were not (t(175) = -1.54, p = .13).

Split half reliability (internal consistency) was 0.70 for the RMET, 0.57 for the CET and 0.41 for AET; while Fleiss’ Kappa for inter-rater agreement was: RMET = 0.40; CET = 0.37; AET = 0.41 (fair agreement is 0.21–0.40 and moderate agreement is 0.40–0.60; [34]).

### Partial correlations

Mean accuracy data were calculated per participant, per task, and checked for normality using Shapiro-Wilk tests. Accuracy data for all three Eyes Tasks were non-normal. We therefore applied a Box-Cox transform to these data (λ = 2) and re-tested with Shapiro-Wilk and confirmed that the data were then normally distributed. The correlation between the RMET and CET was positive and very strong after using the AET to control for reasoning linked to physical features (Pr = .59, p<.0001). The partial correlations between the CET and AET when controlling for the RMET (Pr = .18, p = .02), and between the RMET and AET when controlling for CET (Pr = .21, p = .005), were considerably weaker.

### Predictors of eyes task accuracy

Descriptive statistics for all measures additional to eyes tasks are given in S1 Table. Data was summarized, tested for normality and transformed as explained above. To identify the best model predicting performance on each eyes task the "leaps" R package was used to examine all subsets of possible models, from a single predictor variable up to the maximum of 28 predictors: OCI-R score, rSAS score, EQ score, 3 TAS-20 subscales, 4 IRI subscales, 9 SPQ subscales (e.g. [35]), TMT time difference, DOT-A maximum span, 4 pet questionnaire questions, age, gender, and RT for that eyes task). Optimal models were identified based on lowest value of Mallow’s Cp, which is equivalent to the Akaike Information Criterion. The optimum model for RMET accuracy (F(164,11) = 7.06; p<.0001; adjR^2^ = .276) contained significant predictors RT, ‘pet now’, liking dogs, DOT-A, IRI FS, IRI EC, EQ, SPQ UPE (cognitive perceptual factor) and SPQ ESA (social-interpersonal factor). The best model for CET accuracy (F(169,6) = 5.04; p<.0001; adjR^2^ = .122) contained significant predictors ‘pet now’, disliking cats, liking dogs and IRI FS. Finally, the model for AET accuracy (F(169,6) = 5.89; p<.0001; adjR^2^ = .143) contained significant predictors RT, liking dogs, IRI FS and TAS DDF.

### Post hoc analysis involving predictors of eyes test performance

Liking dogs was predictive of accuracy scores on all three eyes tasks and disliking cats was also predictive of CET scores. Questionnaire data are shown in S2 Table and frequency tables for liking cats or dogs are shown in S3 Table. We therefore conducted two additional regressions using the method described above (DV: dog/cat liking; IVs: age, gender, executive, empathy and clinical measures). Greater dog liking (F(167,8) = 4.20; p = .0001; adjR^2^ = .128) was predicted by having a pet now, lower TAS DIF, lower OCI-R, lower SPQ NCF, higher SPQ SUS and OEB scores. Greater cat liking (F(169,6) = 6.97; p<.0001; adjR^2^ = .17) was predicted by having a pet now, higher IRI PT, lower TAS DIF and higher TAS DDF.

### Replication of core findings

We collected extra data on the new CET (18 trials) and RMET in an additional sample of 228 undergraduate Psychology students (58 males and 170 females; mean age 19.87 years, SD = 1.04, range = 18.26–24.07). When using the best subset of 18 trials of the CET (as identified previously), accuracy was 72.45% (SE = 0.93%), and accuracy for the RMET in this new sample was 72.86% (SE = 0.76%). There was no significant difference between the tasks for accuracy (paired t(227) = -0.97, p = .33). The full correlation between CET and RMET was strong (this new sample: Pr = .38, p<.0001; previous sample for comparison: Pr = .657, p<.0001). When comparing the two CET data samples there was no significant difference for accuracy (t(381) = -1.78, p = .09) or distribution (F(175, 227) = 0.953, p = .74). RMET accuracy (t(73) = -1.38, p = .17) and distribution (F(175, 227) = 1.04, p = .78) were also not significantly different across the two samples.

## Discussion

We aimed to develop a comparison measure for the human RMET using cat eyes, compare performance with the RMET and a matched control task requiring age judgments, and explore factors that may contribute to task performance. Our findings show that healthy participants reach a high degree of consensus when asked to judge the mental state of a cat based on a photograph of its eyes alone, replicated in a second sample. Performance on the CET is also closely related to performance on the RMET. People may have similar perceptions of mental states in cats eyes because they are matching visual cues to a stored template normally used for humans. Indeed, the neural correlates for mental state recognition appear to overlap for humans and non-human animals [36].

Currently owning a pet was predictive of greater accuracy on all both the RMET and CET, suggesting that animal exposure is linked to social cognition. Indeed, previous studies have suggested that owning a companion animal can positively impact empathy and communication abilities [37, 38]. Moreover, we found that greater dog liking predicted greater accuracy on all eyes tasks. One explanation for this relationship could be that greater emotional communication or mental state recognition may occur during interactions between humans and dogs. Interestingly, cat likers reported more difficulty describing feelings and this was not the case for dog likers. Therefore, a tendency towards expressing or communicating emotion could increase both liking dogs and accuracy on eyes tasks.

While mental state recognition from eyes was positively associated with liking dogs (and not liking cats, for the CET), a tendency towards abstract perspective taking was positively associated with liking cats. Cat likers may therefore show a preference for mental state reasoning based on verbal or semantic information, whereas dog likers may respond better to visual social cues. Visual recognition of emotional facial expressions is thought to involve mirroring [39], so dog as opposed to cat liking may reflect tendencies towards mirroring versus mentalizing [40]. Our finding that cat likers seem more oriented towards internal experiences and dog likers appear more emotionally expressive may be in accordance with previous studies suggesting that extraversion is associated with a preference for dogs, whereas introversion and neuroticism is associated with a preference for cats [41, 42].

How can we explain the link between liking dogs and performance on the AET? Although age judgment is non-social, the task still involves appraisal of eyes which have strong social salience. Perhaps liking dogs could predict attention towards eyes, or comfort with eye contact which is needed for careful visual analysis and good performance on all three eyes tasks. RMET performance is typically impaired in ASD, however, given that those with ASD attend more to the faces and eyes of animals than of humans [15, 16], and animal interaction may enhance social skills in people with ASD [43], these individuals could respond differently to the newly developed CET.

Another point to take into account is that accuracy on the eyes tasks reflect consensus. Therefore people who fit the group norm will score highest. Previous studies have linked cat or dog preference to personality [41, 42], which may in turn influence CET performance. Perhaps the degree of liking dogs could be indicative of a tendency towards more of a ‘group mentality’ and social consensus, whereas cat lovers may be more independently minded (like their cats) and therefore less concerned about social norms. Having said this, participants did make their judgments independently and would have been unaware of the likely group consensus during testing. It would be interesting to further test the social cognitive skills of people with a strong liking for either cats, or dogs. One may even speculate that the everyday quality of social interaction experienced by an individual (including with animals) could be reflected in resting state or event-related brain activity in addition to behavioural performance on tasks.

In relation to associations between eyes tasks and other measures, executive functions were not related to CET or AET performance, but working memory predicted RMET accuracy. Correlations between RMET accuracy, schizotypal personality characteristics, and empathy support previous research [24, 27]. No measures of psychopathology were significantly associated with performance on the CET. However, IRI fantasy subscale scores (which assess the tendency to take the perspective of a fictional character) were also related with performance on all three eyes tasks, suggesting that some form of perspective taking is involved in the CET and AET. Overall our findings support the possibility that the CET and AET comprise useful counterpart or control tasks when administered with the RMET, especially in participants with working memory impairment or psychiatric disorders. Social cognitive tasks using non-human stimuli provide a complimentary approach when investigating social cognition, especially in clinical populations. Future evaluation could support the possibility that the CET is less affected by confounds and help interpretation of the basis of task impairments. For example, performance on the CET and the RMET may dissociate in groups who experience some aversive response to human stimuli or human eye gaze (e.g. ASD, social anxiety disorder, trauma etc.).

Although we have confirmed our initial findings in a second sample within this study, further research should continue to refine the CET and AET, particularly to improve internal consistency. Indeed, previous studies have reported poor internal consistency in relation to the RMET, and that it may not meet assumptions of normality [24, 44]. Gender has also been suggested to be potentially influential in terms of RMET performance (e.g. females can show superior performance [45, 46], but see e.g. Baron-Cohen et al. [47] and Cook and Saucier [48]). Our sample was majority female, which could limit generalisability i.e. the present outcome is limited, since many more females were involved. Our findings could also have been influenced by the use of a student sample or differences in presentation formats of the eyes task (we used computerised presentation). We used the response options selected by the majority of participants as our correct answers for the CET and AET, whereas the correct answers for the RMET were determined by selection by at least 5 of 8 experts in the original study. Although these methods are not equivalent, criteria for selecting experts could introduce bias, and we have shown that the correct answers for the RMET would be the same when applying the method used in the current study. Another limitation is that although there was a high degree of consensus within these strongly correlated eyes tasks, and previous studies have shown that RMET performance is correlated with measures of intelligence [49], we cannot know exactly what is being measured. This may become clearer through the application of fMRI. In addition, we also cannot know whether the recognised cat mental states are simply in the eye of the beholder; but then this is also the case for the RMET as we cannot be sure what the people in the photographs were actually thinking or feeling. It is also the case that not all tasks were counterbalanced, and eyes test response options always appeared in a fixed location around the images (as in the standard RMET), so this could be manipulated in future research. Finally, the pet questionnaire was quite crude, and a more fine grained assessment relating to animal contact and preferences, and in tendencies towards anthropomorphism, may yield further insight.

In conclusion, people appear to be able to read the mind in the eyes of a cat, reaching a high level of consensus approaching that for human stimuli. This ability is not influenced by factors such as working memory, schizotypal personality or empathy towards humans, which can predict performance on the human RMET. Liking dogs may predict greater accuracy on tests of social cognition involving facial features. While the CET should be further developed and replicated in additional samples, our findings suggest that future studies should explore the use of similar measures in groups with established impairments in social cognition, given that the ability to apply complex mental states to humans versus non-humans may be differentially affected.

## Supporting information

S1 Fig Example items where greater consensus was reached for the correct answer for cat versus human mental state (‘defiant’: Cat Eyes Test = 75% consensus, Reading the Mind in the Eyes Test = 37%; ‘reflective’: Cat Eyes Test = 63% consensus, Reading the Mind in the Eyes Test = 54%). RMET stimuli freely available online (https://www.autismresearchcentre.com/arc_tests) and cat images online, CC0 public domain (https://picryl.com/media/cat-maine-coon-cats-eyes-animals-c204f0; https://pxhere.com/en/photo/499860).
(DOCX)

S2 Fig (JPG)

S1 Table Descriptive statistics for all tasks and scales. (DOCX)

S2 Table Predictors of accuracy on each eyes task. (DOCX)

S3 Table Frequency tables for ratings reflecting the liking of dogs or cats. (DOCX)
