# Supplementary material for: Predictors of performance on the Reading the Mind in the Eyes Test
Source: PLoS One. 2020 Jul 23;15(7):e0235529. doi: 10.1371/journal.pone.0235529 (PMC7377373; doi:10.1371/journal.pone.0235529)
Supplement: S1 Fig — RMET stimuli freely available online (https://www.autismresearchcentre.com/arc_tests) and cat images online, CC0 public domain (https://picryl.com/media/cat-maine-coon-cats-eyes-animals-c204f0; https://pxhere.com/en/photo/499860). (DOCX) [file pone.0235529.s001.docx]

**
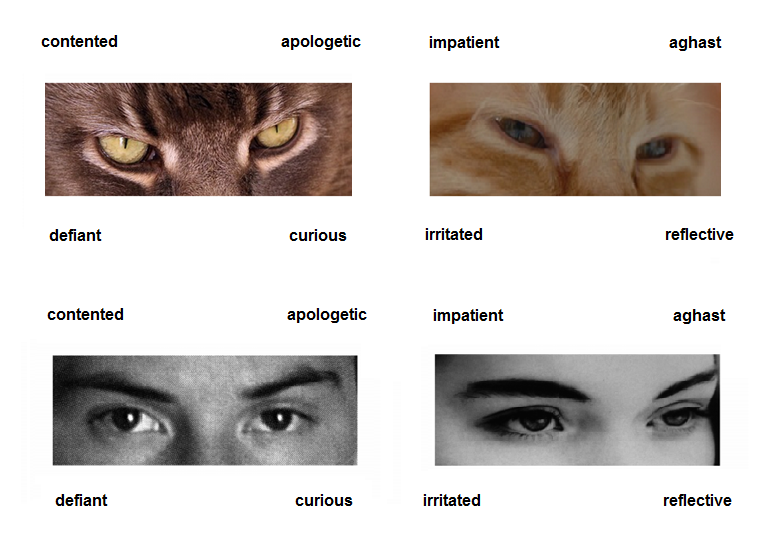
**

**Supplementary Fig 1. Example items where greater consensus was reached for the correct answer for cat versus human mental state.** Trials shown are 'defiant' (CET=75% consensus, RMET=37%) and 'reflective' (CET=63% consensus, RMET=54%). RMET stimuli freely available online (https://www.autismresearchcentre.com/arc_tests) and cat images online, CC0 public domain (https://picryl.com/media/cat-maine-coon-cats-eyes-animals-c204f0; https://pxhere.com/en/photo/499860).
