# Supplementary figures and images for: Predictors of performance on the Reading the Mind in the Eyes Test
Source: PLoS One. 2020 Jul 23;15(7):e0235529. doi: 10.1371/journal.pone.0235529 (PMC7377373; doi:10.1371/journal.pone.0235529)

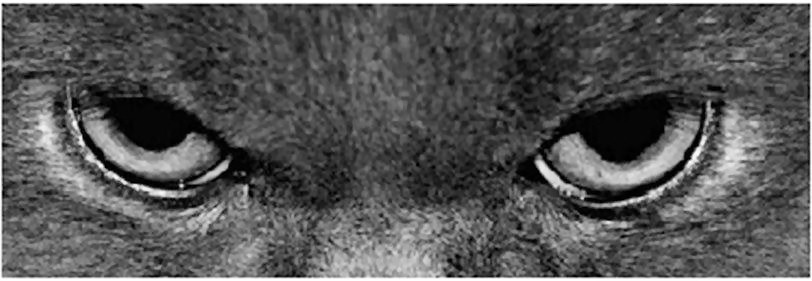

Supplement: S2 Fig — (JPG) [file pone.0235529.s002.jpg]
