# Supplementary material for: Predictors of performance on the Reading the Mind in the Eyes Test
Source: PLoS One. 2020 Jul 23;15(7):e0235529. doi: 10.1371/journal.pone.0235529 (PMC7377373; doi:10.1371/journal.pone.0235529)
Supplement: S1 Table — (DOCX) [file pone.0235529.s003.docx]

**S1 Table.** Descriptive statistics for all tasks and scales

| Task | | | | Subscales | | | | Mean | | | | Standard error | | | | Median | | | | Range | | | |
| --- | --- | --- | --- | --- | --- | --- | --- | --- | --- | --- | --- | --- | --- | --- | --- | --- | --- | --- | --- | --- | --- | --- | --- |
| Pet questionnaire (min -3 to max +3) | | | | Pet now | | | | 0.53 | | | | 0.04 | | | | 1 | | | | 0-1 | | | |
|  |  |  |  | Petprev | | | | 0.84 | | | | 0.03 | | | | 1 | | | | 0-1 | | | |
|  |  |  |  | Liking Cats | | | | 1.77 | | | | 0.10 | | | | 2 | | | | -3 - 3 | | | |
|  |  |  |  | Liking dogs | | | | 2.13 | | | | 0.09 | | | | 3 | | | | -3 - 3 | | | |
| Executive tasks | | | | DOTA (min 2.5; max 8) | | | | 5.64 | | | | 0.08 | | | | 5.50 | | | | 3-8 | | | |
|  |  |  |  | TMT | | | | 34.54 | | | | 2.68 | | | | 26.43 | | | | -15.11-392.00 | | | |
| Interpersonal Reactivity Index (min 28 max 140; per subscale from 7 to 35) | | | | FS | | | | 24.85 | | | | 0.40 | | | | 25.50 | | | | 12-35 | | | |
|  |  |  |  | PT | | | | 26.40 | | | | 0.31 | | | | 27.00 | | | | 16-35 | | | |
|  |  |  |  | PD | | | | 20.47 | | | | 0.35 | | | | 20.00 | | | | 10-33 | | | |
|  |  |  |  | EC | | | | 28.28 | | | | 0.27 | | | | 28.00 | | | | 15-35 | | | |
| Toronto Alexithymia Scale (min 20 max 100) | | | | Total (min/max) | | | | 45.96 | | | | 0.77 | | | | 45.00 | | | | 29-76 | | | |
|  |  |  |  | DIF (7/35) | | | | 15.03 | | | | 0.37 | | | | 14.00 | | | | 2-28 | | | |
|  |  |  |  | DDF (5/25) | | | | 13.13 | | | | 0.33 | | | | 13.00 | | | | 5-25 | | | |
|  |  |  |  | EOT (8/40) | | | | 17.80 | | | | 0.31 | | | | 18.00 | | | | 8-28 | | | |
| Schizotypal Personality Questionnaire (min 0 max 74) | | | | Total (max) | | | | 19.47 | | | | 0.82 | | | | 18.00 | | | | 1-54 | | | |
|  |  |  |  | IOR (9) | | | | 2.59 | | | | 0.18 | | | | 2.00 | | | | 0-9 | | | |
|  |  |  |  | ESA (8) | | | | 4.38 | | | | 0.19 | | | | 5.00 | | | | 0-8 | | | |
|  |  |  |  | OBM (7) | | | | 0.62 | | | | 0.08 | | | | 0.00 | | | | 0-5 | | | |
|  |  |  |  | UPE (9) | | | | 1.73 | | | | 0.14 | | | | 1.00 | | | | 0-9 | | | |
|  |  |  |  | OEB (7) | | | | 1.42 | | | | 0.14 | | | | 0.50 | | | | 0-7 | | | |
|  |  |  |  | NCF (9) | | | | 1.74 | | | | 0.14 | | | | 1.00 | | | | 0-8 | | | |
|  |  |  |  | OS (9) | | | | 3.42 | | | | 0.16 | | | | 3.00 | | | | 0-8 | | | |
|  |  |  |  | CA (8) | | | | 1.53 | | | | 0.12 | | | | 1.00 | | | | 0-7 | | | |
|  |  |  |  | SUS (8) | | | | 2.03 | | | | 0.16 | | | | 2.00 | | | | 0-8 | | | |
| Obsessive Compulsive Inventory Revised (min 0 max 72) | | | | | | | | 14.77 | | | | 0.83 | | | | 11.00 | | | | 0-59 | | | |
| Empathy Quotient (min 0 max 80) | | | | | | | | 46.44 | | | | 0.84 | | | | 46.00 | | | | 10-74 | | | |
| Revised Social Anhedonia Scale (min 0 max 40) | | | | | | | | 6.32 | | | | 0.43 | | | | 4.50 | | | | 0-29 | | | |
| Eyes test accuracy (/1) | | | AET | | | | | 0.58 | | | | 0.01 | | | | 0.57 | | | | 0.32-0.80 | | | |
|  |  |  | RMET | | | | | 0.71 | | | | 0.01 | | | | 0.73 | | | | 0.31-0.94 | | | |
|  |  |  | CET | | | | | 0.58 | | | | 0.01 | | | | 0.59 | | | | 0.30-0.79 | | | |
| Eyes test RT (sec) | | | AET | | | | | 3.58 | | | | 0.08 | | | | 3.50 | | | | 1.18-7.71 | | | |
|  |  |  | RMET | | | | | 4.47 | | | | 0.12 | | | | 4.20 | | | | 1.14-11.71 | | | |
|  |  |  | CET | | | | | 4.35 | | | | 0.10 | | | | 4.21 | | | | 1.58-11.16 | | | |
| Pet Questionnaire (count/n) | Pet now | | | | Pet prev | | How much like cats (+3= I love) | | | | | | | | | How much like dogs (-3=I hate) | | | | | | | |
|  | Y | N | | | Y | N | -3 | | -2 | -1 | 0 | | 1 | 2 | 3 | -3 | -2 | -1 | 0 | | 1 | 2 | 3 |
|  | 92 | 80 | | | 145 | 27 | 1 | | 6 | 7 | 11 | | 35 | 45 | 67 | 1 | 4 | 3 | 6 | | 25 | 43 | 90 |

**KEY:** DOTA: Digit Ordering Test-Adapted; TMT: Trail Making Test; FS: Fantasy; PT: perspective taking; PD: Personal distress; EC: Empathic concern; DIF: Difficulty identifying feelings; DDF: Difficulty describing feelings; EOT: Externally oriented thinking; IOR: Ideas of reference; ESA: Extreme social anxiety; OBM: Odd behaviour and mannerisms; UPE: unusual perceptual experiences; OEB: Odd or eccentric beliefs; NCF: No close friends; OS: Odd speech; CA: Constricted affect; SUS: Suspiciousness; RMET: Reading the Mind in the Eyes Task; CET: Cat Eyes Task; AET: Age Eyes Task; RT: reaction time; Y: Yes; N: No; Pet prev: Pet previously.
