# Supplementary material for: Predictors of performance on the Reading the Mind in the Eyes Test
Source: PLoS One. 2020 Jul 23;15(7):e0235529. doi: 10.1371/journal.pone.0235529 (PMC7377373; doi:10.1371/journal.pone.0235529)
Supplement: S2 Table — (DOCX) [file pone.0235529.s004.docx]

**S2_Table**. Predictors of accuracy on each eyes task

| Task | *Predictors* | *Estimates* | *std. Error* | *CI* | *Statistic* | *p* |
| --- | --- | --- | --- | --- | --- | --- |
| RMET | RMET RT | 0.02 | 0.01 | 0.01 – 0.03 | 3.31 | **0.001** |
|  | PETNOW | 0.06 | 0.02 | 0.02 – 0.09 | 3.42 | **0.001** |
|  | Liking dogs (rating) | 0.02 | 0.01 | 0.00 – 0.03 | 2.43 | **0.016** |
|  | DOTA (working memory) | 0.02 | 0.01 | 0.01 – 0.04 | 3.04 | **0.003** |
|  | IRI Fantasy scale | 0.00 | 0.00 | 0.00 – 0.01 | 2.66 | **0.009** |
|  | IRI Personal distress | -0.00 | 0.00 | -0.01 – 0.00 | -1.79 | 0.075 |
|  | IRI Empathic concern | -0.01 | 0.00 | -0.01 – -0.00 | -2.73 | **0.007** |
|  | TAS Externally oriented thinking | -0.00 | 0.00 | -0.01 – 0.00 | -1.46 | 0.145 |
|  | SPQ Excessive social anxiety | 0.01 | 0.00 | 0.00 – 0.02 | 2.72 | **0.007** |
|  | SPQ Unusual perceptual experiences | -0.01 | 0.00 | -0.02 – -0.00 | -2.07 | **0.040** |
|  | EQ | 0.00 | 0.00 | 0.00 – 0.00 | 2.49 | **0.014** |
| AET | AET RT | 0.02 | 0.01 | 0.01 – 0.04 | 2.84 | **0.005** |
|  | Liking cats (rating) | -0.01 | 0.01 | -0.02 – 0.00 | -1.78 | 0.077 |
|  | Liking dogs (rating) | 0.03 | 0.01 | 0.01 – 0.04 | 3.77 | **<0.001** |
|  | IRI Fantasy scale | 0.00 | 0.00 | 0.00 – 0.01 | 2.20 | **0.029** |
|  | TAS Difficulty defining feelings | 0.00 | 0.00 | 0.00 – 0.01 | 1.99 | **0.048** |
|  | SPQ suspiciousness | -0.01 | 0.00 | -0.01 – 0.00 | -1.81 | 0.073 |
| CET | CET RT | 0.01 | 0.01 | -0.00 – 0.03 | 1.90 | 0.059 |
|  | PETNOW | 0.06 | 0.02 | 0.02 – 0.10 | 2.70 | **0.008** |
|  | Liking cats (rating) | -0.02 | 0.01 | -0.03 – -0.00 | -2.50 | **0.014** |
|  | Liking dogs (rating) | 0.02 | 0.01 | 0.00 – 0.03 | 2.09 | **0.038** |
|  | IRI Fantasy scale | 0.00 | 0.00 | 0.00 – 0.01 | 2.39 | **0.018** |

KEY: AET: Age eyes task; CET: Cat eyes task; DOT-A: Digit Ordering Test-Adapted; EQ: Empathy Quotient; IRI: Interpersonal Reactivity Index; PETNOW; rating of whether have a pet currently; RMET; Reading the Mind in the Eyes Test; RT: Reaction time; SPQ; Schizotypal Personality Questionnaire; TAS: Toronto Alexithymia Scale.
