# Supplementary material for: Predictors of performance on the Reading the Mind in the Eyes Test
Source: PLoS One. 2020 Jul 23;15(7):e0235529. doi: 10.1371/journal.pone.0235529 (PMC7377373; doi:10.1371/journal.pone.0235529)
Supplement: S3 Table — (DOCX) [file pone.0235529.s005.docx]

**S3_Table**. Frequency tables for ratings reflecting the liking of dogs or cats

| Liking Cats rating | Liking Dogs rating | | | | | | |
| --- | --- | --- | --- | --- | --- | --- | --- |
|  | -3 (I hate) | -2 | -1 | 0 | 1 | 2 | 3 (I love) |
| -3 | 0 | 0 | 1 | 0 | 0 | 0 | 0 |
| -2 | 0 | 0 | 1 | 1 | 1 | 0 | 1 |
| -1 | 0 | 1 | 0 | 0 | 0 | 0 | 2 |
| 0 | 0 | 0 | 0 | 0 | 1 | 4 | 1 |
| 1 | 0 | 0 | 1 | 2 | 5 | 7 | 10 |
| 2 | 0 | 3 | 2 | 4 | 12 | 6 | 16 |
| 3 | 1 | 2 | 2 | 4 | 16 | 28 | 37 |
